# Supplementary material for: Ammonia-oxidizing archaea possess a wide range of cellular ammonia affinities
Source: ISME J. 2021 Jul 27;16(1):272–83. doi: 10.1038/s41396-021-01064-z (PMC8692354; doi:10.1038/s41396-021-01064-z)
Supplement: Supplementary file 1 — Supplemental Material [file 41396_2021_1064_MOESM1_ESM.docx]

**This document includes:**

Supplementary Material and Methods

Supplementary Results and Discussion

Supplementary Tables S1 to S4

Supplementary Figures S1 to S8

Supplementary References

**Supplementary Materials and Methods**

***Isolation of ‘*Candidatus *Nitrosotenuis uzonensis’ N4***

‘*Ca.* N. uzonensis’ N4 was isolated from a previously described thermal spring ammonia-oxidizing enrichment culture [1]. Further enrichment and subsequent purification of ‘*Ca*. N. uzonensis N4’ was performed over the course of 6 years. Successive cultures were maintained in a mineral salt medium (Table S1) containing a mixture of antibiotics (ampicillin and penicillin-G; 50 μg ml^-1^). Cultures were transferred approximately every three weeks, and a pure culture designated strain N4 was obtained through filtration (0.22-μm syringe filter; Sigma Aldrich) and subsequent end-point serial dilution in deep well 96-well plates containing medium supplemented with ammonium chloride (100 μM) and pyruvate (50 μM). The activity of strain N4 was monitored by determining ammonium consumption and nitrite production photometrically [2-5]. After isolation, ‘*Ca*. N. uzonensis’ N4 was routinely grown in pyruvate supplemented medium without antibiotics. Cultures were maintained in the dark, without shaking at 37ºC. A general bacterial 16S rRNA gene PCR (27F and 1492R) [6] was used to verify the absence of any bacterial contaminants as described in Weisburg *et al*.,[7]. Culture purity was confirmed and routinely monitored by fluorescence *in situ* hybridization (FISH) as previously described [8] with a Cy3-labeled *Archaea*-specific probe (Arc915) [9] and a FAM-labeled *Bacteria*-specific probe (EUB338) [10]. To visualize all cells, 4',6-diamidino-2-phenylindole (DAPI) staining was used. In addition, the absence of bacterial growth in three nutrient-rich media broths was routinely observed (i.e., lysogeny broth, Reasoners 2A broth, and tryptic soy broth) [11].

***New thermophilic AOA enrichment cultures***

Cultivation of ‘*Ca.* Nitrosocaldus yellowstonensis’ HL72 (formerly published as ‘*Ca.* N. yelllowstonii’ HL72) from hot spring sediment from Yellowstone National Park, Wyoming (USA) was previously described [12]. Two additional thermophilic AOA cultures were enriched using the same medium and approach used to cultivate HL72. ‘*Ca*. Nitrosofervidus tenchongensis’ DRC1 was cultivated at 72˚C from the Direchi Geothermal Pond (地热池) in the Rehai geothermal field in Tengchong, Yunnan Province, China. The temperature and pH at the sampling site (N 24.95009°, E 98.43807°) were 83˚C and pH 8.3, respectively. Additional geochemical characterization of the site has been described elsewhere [13, 14]. ‘*Ca*. Nitrososphaera nevadensis’ GerE is a moderately thermophilic strain growing at 50˚C and was cultivated from a low temperature spring (45˚C, pH 8.0) located adjacent to Great Boiling Spring (N 40.66148˚, E 119.36613˚) in the Great Basin of Nevada (USA) [15].

***Cultivation conditions of ammonia-oxidizing microorganisms***

The medium description and cultivation conditions of all ammonia-oxidizing microorganisms (AOM) used in this study are provided in Tables S1 and S2. Briefly, all AOM cultures were grown in Schott bottles at their optimal temperature and pH, in the dark, without shaking, unless otherwise noted. When necessary, the initial pH of all AOM media was adjusted with 1 M HCl and 1 M NaOH. Substrate was provided in the form of NH_4_Cl from pre-sterilized stocks and replenished as necessary. Sterile sodium bicarbonate (1M) was added as necessary in order to adjust culture pH. An initial concentration of 0.5 mM sodium pyruvate was added to the cultivation medium of *N. koreense* MY1, ‘*Ca.* N. chungbukensis’ MY2, ‘*Ca.* N. uzonensis’ N4, *N. maritimus* SCM1, *N. piranensis* D3C, and *N. adriaticus* NF5 as a hydrogen peroxide (H_2_O_2_) scavenger [16].

***Nitrite-dependent ammonia oxidation kinetics***

In addition to microrespirometry measurements, ammonia oxidation kinetics of ‘*Ca*. Nitrosocosmicus franklandus’ C13 were also determined by nitrite accumulation activity assays performed with concentrated cell suspensions as previously described [17]. Briefly, ‘*Ca*. Nitrosocosmicus franklandus’ C13 was grown in batch culture until mid-exponential growth phase was reached (corresponding to 600-700 μM nitrite accumulated in the medium). Cells from 1.6 L of culture were harvested onto 0.22 μm pore size, 45 mm diameter polyethersulfone (PES) filter (PALL). Cells were washed with 10 mM HEPES (pH 7.3)-buffered medium with no added ammonium to remove residual ammonium and nitrite. Cells were then resuspended in 200 mL 10 mM HEPES (pH 7.5)-buffered medium with no added ammonium. Concentrated cells were incubated at 37°C without ammonium for 60 min to allow the endogenous respiration to cease. For cell counts, cells were stained with DAPI and counted on 0.22 μm pore size black polycarbonate filters using fluorescence microscopy as previously described [18].

For the kinetics assays, cell concentrations were adjusted to approximately 1 × 10^7^ ml^-1^ to ensure that the rates of nitrite accumulation were always linear over the course of the entire assay. Aliquots of 5 ml cell suspension were added to acid-washed 23 ml glass vials which were sealed with twice autoclaved grey butyl rubber septa. The assays were performed in a 37°C or 42°C static water bath and all treatments were performed in triplicate vials. Ammonia-oxidizing activity was initiated by adding NH_4_Cl at the following final concentrations: 5, 10, 25, 50, 100, 250, 500, and 1000 μM. 100 μl volume was removed from each vial at 15 min intervals using a needle and syringe, and nitrite concentration was measured immediately after sampling. Assays were run over a period of 60 min. *K*_m(app)_ and *V*_max_ values were determined using the Hyper32 kinetics package (Hyper32.exe, version 1.0.0, 2003). Protein determination was carried out using the PierceTM BCA Protein Assay Kit (ThermoFisher Scientific) according to the manufacturer’s instructions.

Nitrite concentrations were determined colorimetrically using the Griess reagent in 96-well flat bottom clear microtiter plates as previously described [18]. Briefly, 20 μl sulphanilamide solution (5 g L^-1^ in 2.4 M HCl) was added to 100 μl of sample or standard, followed by the addition 20 μl of naphthylethylenediamide solution (3 g L^-1^ in 0.12 M HCl). Standards were performed in duplicate, prepared using KNO_2_ and ranging from 0.5 to 50 μM. Absorbance was recorded at a wavelength of 540 nm using a VersaMax platereader (Molecular Devices, CA, US).

***Statistical analysis***

Statistical analyses were performed using the R environment for statistical computing (http://www.R-project.org/) and SigmaPlot 11.0 (Systat Software Inc., San Jose, CA, USA). An independent-sample *t*-test was performed to assess the significant difference between two groups, and *p* values less than 0.05 were considered to be statistically significant.

**Supplementary Results and Discussion**

***Cellular kinetic properties across studies***

**The effect of differential medium conditions on AOM cellular kinetic properties.**

Differences in growth medium composition may affect the physiological state of AOM, potentially leading to unreproducible cellular kinetic measurements. To test this hypothesis, the cellular kinetic properties of *N. inopinata* were measured under optimal growth temperature and pH, in a different growth medium than used previously for the kinetic characterization of this organism [19]. In these experiments we determined only a minute effect on the *K*_m(app)_ for NH_3,_ *K*_m(app)_ for total ammonium (NH_3_ + NH_4_^+^), or *V*_max_, and no significant effect on the *a*^o^ for NH_3_ or total ammonium (NH_3_ + NH_4_^+^) (*p* > 0.7) (Fig. 2, Figs. S2 and S8). In addition, the substrate affinity for NH_3_ of *Nitrosomonas europaea* (*K*_m(app)_=23.3 µM, s.d.=1.5; Fig. S8) and *Nitrosarchaeum koreense* MY1 (*K*_m(app)_=4.8 nM, s.d.=0.5; Figs. S1 and S2) which were previously determined in different media, were confirmed in this study. Notably, there was no significant difference (*p* = 0.74) between the cellular kinetics of *N. koreense* MY1 when determined as an enrichment culture (containing <10% partner bacteria) [20] or as pure culture in this study (Fig. S2). This indicates that the different growth media used to culture AOM in this study, does not affect the reproducibly of the cellular kinetic property measurements.

**Activity versus growth kinetic properties**

The substrate affinity of *N. maritimus* SCM1 for NH_3_ was reported to be ~3 nM, as determined by substrate-dependent oxygen uptake measurements, which was about three orders of magnitude higher than the affinity for NH_3_ of any characterized ammonia-oxidizing bacterium (AOB) [21]. Based on this analysis of *N. maritimus*, it was widely generalized in many studies that AOA possess a high affinity for NH_3_, and interpreted as an explanation for the high abundance of AOA compared with AOB in nutrient poor environments [22] until it was determined that non-marine AOA do not all possess such a high affinity for NH_3_ [19].

Recently, the primary report of the substrate affinity of *N. maritimus* SCM1 for NH_3_ [21] was called into question [23], and the revised substrate affinity of *N. maritimus* SCM1 was reported to be ~2 µM. This represents a substrate affinity three orders of magnitude lower than previously determined, and within the same range as the substrate affinity of the AOB *Nitrosomonas europaea* - an AOB adapted to high substrate concentrations [23]. Both sets of kinetic experiments were performed in the same growth medium and at the same temperature (30°C). In addition, the strains used displayed near-identical maximum specific growth rates (0.027 versus 0.028 h^-1^) in batch culture [23]. However, in Hink *et al*. [23], kinetic parameters were determined from the activity of an actively growing culture that was shaking over the course of about 10 days. In contrast, in Martens-Habbena *et al*. [21], kinetic parameters were determined from a culture that was grown statically, although the culture was stirred over the course of a few hours during the instantaneous activity measurements. These differences in methodology most likely play a large role in the observed differences in kinetic parameters between the two studies, as several studies have previously reported that *N. maritimus*, and ammonia oxidizers in general, are sensitive to continuous shaking [20, 21, 24]. Finding an explanation of these sharply contrasting observations is important, not just for defining the kinetic characteristics of *N. maritimus* SCM1, but for how widely applicable and reproducible these types of kinetic studies are.

It was speculated that *N. maritimus* SCM1 may have become domesticated and lost its high substrate affinity during years of laboratory cultivation at an increased substrate concentration, as the selective pressure which it was under in the environment was removed. To test this hypothesis, we investigated the kinetic properties of the *N. maritimus* SCM1 strain used in Hink *et al*. [23] (kindly provided by the lab of James I. Prosser), using the microrespirometry (MR) method that was applied in Martens-Habbena *et al*. [21]. As we were able to reproduce the substrate affinity values for *N. maritimus* SCM1 (*K*_m(app)_ = ~3.33 nM for NH_3_) reported by Martens-Habbena *et al*. [21], we can rule out domestication of *N. maritimus* SCM1 as the cause of these contrasting results (Fig. 2, Figs. S1 and S2).

Although we rule out domestication in this specific case, we cannot rule out possible domestication effects of all isolated AOA characterized, as domestication effects are dependent on the number of generations and not strictly from the time of isolation. However, we can report that the initial substrate concentration used during the enrichment process does not correlate with the observed substrate affinities for NH_3_ or total ammonium.

Therefore, we propose that a possible explanation of the observed contrasting results represents the difference between the half saturation activity/growth properties (*K*_m_/*K*_s_) as determined by Michaelis-Menten (S1) and Monod kinetics (S2), respectively:

(S1) *V* = (*V*_max_ × [*S*]) × (*K*_m(app)_ + [*S*])^-1^

(S2) *µ* = (*µ* _max_ × [*S*]) × (*K*_s(app)_ + [*S*])^-1^

Although both equations utilize the same form, the Michaelis-Menten equation model activity whereas the Monod equation models growth. This represents the difference between the *K*_m_ (the half saturation of activity, or the substrate concentration when the activity rate is half of the maximal activity rate) and the *K*_s_ (the half saturation of growth, or the substrate concentration when the growth rate is half of the maximal growth rate). The major difference is that cellular processes involved in microbial growth such as repair, turnover, stress response, and division are accounted for in the Monod but not in the Michaelis-Menten model [25]. With this rationale, the *K*_m(app)_ reported in the growth experiment in Hink *et al*. [23] may actually represent a *K*_s(app)_ value, compared with the *K*_m(app)_ activity values generated in this study and by Martens-Habbena *et al*. [21]. This explanation has recently also been proposed to describe these observed differences, however without experimentally ruling out strain domestication [26]. Even with this explanation, it is still hard to explain why these two kinetic parameters (*K*_m(app)_ and *K*_s(app)_) observed under ideal conditions vary by three orders of magnitude.

**The effect of culture growth stage and temperature on the cellular kinetics of AOM**

The cellular kinetics of the enrichment culture ‘*Ca.* N. uzonensis’ N4 have previously been reported [19]. Since this report, ‘*Ca.* N. uzonensis’ N4 has been isolated (detailed above) and the cellular kinetics were re-determined here using the pure ‘*Ca.* N. uzonensis’ N4 culture. Surprisingly, the substrate affinity of the pure ‘*Ca.* N. uzonensis’ N4 culture was orders of magnitude higher (lower *K*_m(app)_) than what was originally measured for the enrichment culture (Figs. S1 and S2). Notably, the stoichiometry of NH_4_^+^ and O_2_ consumption was close to 1:1.5 (1:1.48, s.d=0.02, *n*=5) in the enrichment culture when measured in 2017. Therefore, a strong microrespirometry bias caused by respiration of heterotrophic contaminants had been ruled out.

One possible explanation for the different results between enrichment and pure culture characterization is the difference in the growth phase of the cultures before the MR experiments. In Kits *et al*. [19] ‘*Ca.* N. uzonensis’ N4 enrichment cultures were harvested from batch cultures at the onset of substrate depletion (onset of stationary phase) without concentration of biomass, as the enrichment completely lost ammonia oxidation activity after concentration attempts by centrifugation. In contrast, in the present study, pure active ‘*Ca.* N. uzonensis’ N4 biomass was successfully concentrated using Amicon filter units (see details in Materials and Methods in the main text). Previously, it has been observed that the substrate affinity of *Nitrobacter winogradsky* differs by more than an order of magnitude between biomass harvested during exponential phase versus stationary phase [27]. In addition, the ‘*Ca.* N. uzonensis’ N4 enrichment culture previously tested in 2017 was re-examined after being cultured at 37°C and harvested using the Amicon filter units. Using these conditions, there was no significant difference (*p* = 0.95) between the substrate affinity for NH_3_ observed between the enrichment culture (*K*_m(app)_=18.05 nM, s.d.=9.55) and the pure culture (*K*_m(app)_=19.1 nM, s.d.=6.98; Fig. S1), but we cannot rule out that the composition of the enrichment culture changed between 2017 and our study.

A second possible explanation for the observed differences between the enrichment culture and the pure culture is an effect of temperature on ‘*Ca.* N. uzonensis’ N4 cellular kinetics, as the measurements were previously performed at 46°C [19], versus 37°C in this study. Indeed, increased substrate affinities at lower temperatures were also observed in the present study for ‘*Ca*. N. oleophilus’ MY3 (Fig. S7) and for *N. viennensis* EN76 (~400 nM at 42 °C versus ~20-60 nM NH_3_ at 30°C) across two independent studies [19, 28]. In the latter example, there were no large differences in growth medium or culture pH, but one study was performed at the optimal growth (determined by nitrite production) temperature of 42°C [19], whereas the other study was performed at the more environmentally relevant temperature of 30°C [28]. However, neither of these examples display the variation observed for ‘*Ca.* N. uzonensis’ N4. Therefore, we postulate that due to the differences in cell harvesting techniques, the reported kinetic properties of ‘*Ca.* N. uzonensis’ N4 are likely representative of different growth stages.

**Determining cellular activity kinetics with MR versus nitrite production**

The cellular kinetics of ‘*Ca*. Nitrosocosmicus franklandus’ C13 were determined in this study by two different methods, both MR and short-term activity (measured by NO_2_^-^ production) (Fig. S2). The MR experiments and short-term activity measurements were performed at the optimal growth temperature of 42°C [18]. Notably, there were significant differences (*p* < 0.01) in the observed *K*_m(app)_ for NH_3_ between the two methods. It is possible this difference stems from the different methods used to harvest the cells or reflects that the cells were in different growth phases when the kinetic experiments were performed. For the MR experiments, ‘*Ca*. Nitrosocosmicus franklandus’ C13 culture was taken without concentration once a culture depleted all the substrate. This means that the growth phase of the culture was late-log or early stationary phase. In contrast, for the short-term activity assays, ‘*Ca*. Nitrosocosmicus franklandus’ C13 cultures were harvested (filtered, see above) when in log phase. While these differences in the *K*_m(app)_ for NH_3_ of ‘*Ca*. Nitrosocosmicus franklandus’ C13 are significant, they are all well within the range of the *K*_m(app)_ for NH_3_ of the other AOA in the *Nitrososphaeraceae* lineage.

Together, these observations highlight the importance of cultivation conditions and the methods used when determining the cellular kinetics of AOM. They raise an interesting question about whether the kinetic properties determined under more *in situ*-like conditions or at laboratory determined optimal conditions provides more useful information. These differences will need to be considered in future studies, as most AOM are cultivated at temperatures much higher than what is found in their natural environmental habitat. In addition, these observations also highlight the fact that the whole cell (apparent) kinetic properties reported here and elsewhere have inherent plasticity and are not equivalent to kinetic constants. This is important to remember when predicting which AOM may outcompete others in both laboratory and environmental settings.

**Supplementary Tables and Figures:**

**Table S1.** Growth medium of the ammonia-oxidizing microorganisms used in this study.

|  | **Medium #1^a^** | **Medium #2^b^** | **Medium #3^c^** | **Medium #4^d^** | **Medium #5^e^** |
| --- | --- | --- | --- | --- | --- |
| **Alternative name** | - | Artificial freshwater medium | Synthetic *Crenarchaeote* medium | - | - |
| **Cultures** | ‘*Ca.* N. uzonensis’ N4  *N. inopinata*  *N. europaea* | ‘*Ca.* N. chungbukensis’ MY2  ‘*Ca.* N. oleophilus’ MY3  ‘*Ca.* N. franklandus’ C13  *N. koreense* MY1 | *N. maritimus* SCM1  *N. piranensis* D3C  *N. adriaticus* NF5 | ‘*Ca*. N. devanaterra’ Nd1  ‘*Ca*. N. sinensis’ Nd2 | *‘Ca*. N. yellowstonensis’ HL72  ‘*Ca*. N. tenchongensis’ DRC1  ‘*Ca*. N. nevadensis’ GerE |
| **Contents**  **(g l^-1^)** | **(g l^-1^)**  NaCl (0.6)  MgSO_4_ × 7 H_2_O (0.05)  KH_2_PO_4_ (0.05)  KCl (0.075) | **(g l^-1^)**  NaCl (1.0)  MgCl_2_ × 6H_2_O (0.4)  CaCl_2_ × 2H_2_O (0.1)  KH_2_PO_4_ (0.2)  KCl (0.5) | **(g l^-1^)**  NaCl (26)  MgSO_4_ × 7H_2_O (5)  MgCl_2_ × 6H_2_O (5)  CaCl_2_ × 2H_2_O (1.5)  Kbr. (0.1) | **(g l^-1^)**  NaCl (1)  MgCl_2_ × 6H_2_O (0.4)  CaCl_2_ × 2H_2_O (0.1)  KH_2_PO_4_ (0.2)  KCl (0.5) | **(g l^-1^)**  NaCl (1)  MgCl_2_ × 6H_2_O (0.4)  CaCl_2_ × 2H_2_O (0.1)  KCl (0.5) |
| **Additional contents**  **(stock solutions)** | **Trace element solution contents (TES) (mg l^-1^):** HCl (37%) (2.5 ml l^-1^)  MnSO_4_ × 1 H_2_O (34.4) H_3_BO_3_ (50.0) ZnCl_2_ (70.0)  Na_2_MoO_4_ × 2 H_2_O(72.6)  CuCl_2_ × 2 H_2_O (20.0)  NiCl_2_ × 6 H_2_O (24.0)  CoCl_2_ × 6 H_2_O (80.0)  FeSO_4_ × 7 H_2_O (1000)  **Selenium wolfram solution (SWS)**  **(mg l^-1^):**  Na_2_SeO_3_ × 5H_2_O (3.0)  Na_2_WO_4_ × 2H_2_O (4.0)  NaOH. (500)  **CaCl_2_ solution (g l^-1^):**  CaCl2 x 2H2O (147) | **FeNaEDTA (2.75 g l^-1^)**  **Non-chelated TES (mg l^-1^):**  HCI (25%) (12.5 ml l^-1^)  FeSO_4_ × 7H20 (2100)  H_3_BO_3_ (30)  MnC1_2_ × 4H_2_0 (100)  CoC1_2_ × 6H_2_0 (190)  NiC1_2_ × 6H_2_0 (24)  CuC1_2_ × 2H_2_0 (2)  ZnSO_4_ × 7H_2_0 (144)  Na_2_MoO_4_ × 2H20 (36)  **Vitamin solution (mg l^-1^):**  Biotin (20)  Folic Acid (20)  Pyridoxine HCl (100)  Thiamine HCl (50)  Riboflavin (50)  Nicotinic Acid (50)  DL Pantothenic Acid (50)  p Aminobenzoic Acid (50)  Lipoic acid (50)  1,4-naphthaquinine (40)  Nicotinamide (100)  Hemin (10)  Vitamin B_12_ (10)  pH =7 with KOH | **TES (see Medium #1)**  **SWS (see Medium #1)**  **HEPES (238 g l^-1^)**  **NaHCO_3_ (84 g l^-1^)**  **KH_2_PO_4_ (0.4 g l^-1^)**  **FeNaEDTA (2.75 g l^-1^)**  **Na-pyruvate (110 g l^-1^)** | **TES (see Medium #1)**  **FeNaEDTA (2.75 g l^-1^)**  **MES hydrate**  **Oxalacetic acid (66 g l^-1^)** | **TES (see Medium #1)**  **SWS (see Medium #1)**  **NaHCO_3_ (84 g l^-1^)**  **MOPS (209.2 g l^-1^) pH7.5**  **KH_2_PO_4_ (136 g l^-1^)**  **FeNaEDTA (2.75 g l^-1^)**  **Vitamin solution (mg l^-1^):**  Biotin (20)  Folic Acid (20)  Pyridoxine HCl (100)  Thiamine HCl (50)  Riboflavin (50)  Nicotinic Acid (50)  DL Pantothenic Acid (50)  p Aminobenzoic Acid (50)  Choline Chloride (2000)  Vitamin B_12_ (10)  pH =7 with KOH |
| **Procedure** | Contents were autoclaved and the additional contents were aseptically added afterwards (ml l^-1^):   - TES (1) - SWS. (1) - CaCl_2_ (1) | Contents were autoclaved and the additional contents were added aseptically afterwards (ml l^-1^):   - Non-chelated TES (1) - Vitamin solution. (1) - FeNaEDTA. (1) - HEPES (10) - NaHCO_3_ (2) | Contents were autoclaved and the additional contents were added aseptically afterwards (ml l^-1^):   - HEPES (10) - NaHCO_3_ (2) - KH_2_PO_4_ (5) - FeNaEDTA (1) - TES (1) - Na-pyruvate (1) | Contents and additional contents were combined and then filter sterilized (0.22µm). The amount of additional contents added were:   - Oxalacetate (1 ml l^-1^) - FeNaEDTA (1 ml l^-1^) - TES (1 ml l^-1^) - MES hydrate (1.95 g) | Contents were autoclaved and the additional contents were added aseptically afterwards (ml l^-1^):   - TES (10) - SWS (1) - NaHCO_3_ (5) - KH_2_PO_4_ (0.3) - FeNaEDTA (1) - Vitamin solution (1) - MOPS. (10) |

^a^Medium was modified from Kits *et al*., 2017 [19].

^b^Medium was modified from Jung *et al*., 2011 [20].

^c^Medium was modified from Könnecke *et al*., 2005 [29].

^d^Medium was modified from Lehtovirta-Morley *et al*., 2011 [30].

^e^Medium was modified from de la Torre *et al*., 2008 [12].

**Table S2**. Optimal growth conditions and the conditions used for cellular kinetic experiments in this study for all tested ammonia-oxidizing microorganisms. References are provided for optimal growth conditions. Cellular kinetics were determined at the temperature and pH ranges noted in parenthesis, if different than optimal growth conditions.

| **Type of nitrifier** | |  |  | **Growth optimum** | |  |  | |  |
| --- | --- | --- | --- | --- | --- | --- | --- | --- | --- |
| **Type** | **Group** | **Culture** | | **pH** | **Temperature (**°C) | **Medium^a^** | **Isolation source** | | **Reference^b^** |
| AOA | *Nitrosopumilales* | *Nitrosopumilus maritimus* SCM1 | Pure | 7.3 | 32 | #3 | Marine aquarium | [29, 31] | |
|  |  |  |  | (8) | (30) |  |  |  |  |
|  |  | *Nitrosopumilus piranensis* D3C | Pure | 7.3 | 30 | #3 | Marine water | | [24] |
|  |  |  |  | (7) | (30) |  |  |  |  |
|  |  | *Nitrosopumilus adriaticus* NF5 | Pure | 7.3 | 30 | #3 | Marine water | | [24] |
|  |  |  |  | (7) | (30) |  |  |  |  |
|  |  | *Nitrosarchaeum koreense* MY1 | Pure | 7.0 | 25 | #2 | Agricultural soil | | [32] |
|  |  |  |  | (7.2) | (25) |  |  |  |  |
|  |  | ‘*Ca.* Nitrosotenuis chungbukensis’ MY2 | Pure | 7.0 | 30 | #2 | Agricultural soil | | [33] |
|  |  |  |  | (7.3) | (30) |  |  |  |  |
|  |  | ‘*Ca.* Nitrosotenuis uzonensis’ N4^c^ | Pure | 7.5 | 37 | #1 | Thermal spring | | [1], this study |
|  |  |  |  | (7.1-7.4) | (37) |  |  |  |  |
|  |  |  | Enrichment | (7.75) | (37) |  |  |  |  |
|  | *‘Ca.* Nitrosotaleales’ | ‘*Ca.* Nitrosotalea devanaterra’ Nd1 | Pure | 5.3 | 30 | #4 | Acidic soil | | [34] |
|  |  | ‘*Ca.* Nitrosotalea sinensis’ Nd2 | Pure | 5.3 | 35 | #4 | Acidic soil | | [34] |
|  | *Nitrososphaerales* | *Nitrososphaera viennensis* EN76 | Pure | 7.6 | 42 | -^d^ | Garden soil | | [35, 36] |
|  |  | ‘*Ca.* Nitrososphaera gargensis’ | Pure | 7.5 | 42 | -^d^ | Thermal spring | | [37] |
|  |  | ‘*Ca.* Nitrosocosmicus oleophilus’ MY3 | Pure | 7.0 | 30 | #2 | Oil contaminated sediment | | [38] |
|  |  |  |  | (6.5-8.3) | (30) |  |  |  |  |
|  |  | ‘*Ca.* Nitrosocosmicus franklandus’ C13 | Pure | 7.8 | 42 | #2 | Agricultural soil | | [18] |
|  |  |  |  | (7.1-8) | (37-42) |  |  |  |  |
|  |  | *‘Ca.* Nitrososphaera nevadensis’ GerE^3^ | Enrichment | 7.1 | 50 | #5 | Thermal spring | | This study |
|  |  |  |  | (7.35-7.5) | (50) |  |  |  |  |
|  | ‘*Ca*. Nitrosocaldales’ | ‘*Ca.* Nitrosothermus tengchongensis’ DRC1^3^ | Enrichment | 7.1 | 72 | #5 | Thermal spring | | This study |
|  |  |  |  | (7.3-7.5) | (72) |  |  |  |  |
|  |  | ‘*Ca.* Nitrosocaldus yellowstonensis’ HL72^3^ | Enrichment | 7.1 | 72 | #5 | Thermal spring | | [12] |
|  |  |  |  | (7.3-7.5) | (72) |  |  |  |  |
| Comammox | Clade A | *Nitrospira inopinata* | Pure | 7.5 | 37 | #1 | Thermal spring | | [19] |
|  |  |  |  | (6.7-8.4) | (37) |  |  |  |  |
| AOB | Beta-proteobacteria | *Nitrosomonas europaea* ATCC19718 | Pure | 7.5 | 25 | #1 | Soil | | [20, 27] |

^a^Medium contents are described in Table S1.

^b^References refer to the physiological characterization of optimum growth pH and temperature for each AOM strain.

^c^The pH and temperature optimum have not been previously reported for these AOM strains.

^d^*N. gargensis* and *N. viennensis* were not analyzed in this study, but are included here for comparison.

**Table S3.** The effect of medium pH and temperature on the cellular kinetic properties of selected ammonia-oxidizing microorganisms.

| **Culture** | **Temperature (°C)** | **Cell preparation** | **pH** | **Number of replicates** | **Average measured *K*_m(app)_ for NH_3_ + NH_4_^+^ (µM)** | **Average calculated *K*_m(app)_ for NH_3_ (nM)** | ***V*_max_ (μmol N mg protein^-1^ h^-1^)^a^** | **Reference** |
| --- | --- | --- | --- | --- | --- | --- | --- | --- |
| ‘*Ca.* Nitrosocosmicus oleophilus’ MY3^b^ | 30 | Whole-cell | 6.5 | 7 | 2516.87 ± 647.78 | 6630.11 ± 935.50 | 37.04 ± 6.41 | This study |
|  |  |  | 7.2 | 6 | 818.73 ± 185.44 | 9792.16 ± 1322.34 | 36.12 ± 5.13 |  |
|  |  |  | 8.3 | 5 | 246.29 ± 97.82 | 26284.80 ± 3460.81 | 35.84 ± 5.01 |  |
|  | 25 |  | 7.2 | 2 | 879.23 ± 10.98 | 7953.70 ± 98.11 | 21.97 ± 1.32 |  |
|  | 30 |  |  | 2 | 970 ± 19.42 | 12233 ± 244.63 | 35.11 ± 0.11 |  |
|  | 35 |  |  | 2 | 832.42 ± 15.01 | 14607.28 ± 264.41 | 47.75 ± 2.88 |  |
| *Nitrospira*  *inopinata*^b^ | 37 | Whole-cell | 6.7 | 7 | 2.24 ± 0.53 | 12.32 ± 1.55 | 12.35 ± 0.46 | This study |
|  |  |  | 7.1 | 4 | 0.97 ± 0.17 | 15.47 ± 2.69 | 14.77 ± 1.35 |  |
|  |  |  | 7.8 | 5 | 0.39 ± 0.09 | 30.33 ± 7.61 | 10.42 ± 2.09 |  |
|  |  |  | 8.4 | 5 | 0.24 ± 0.08 | 36.99 ± 12.12 | 10.56 ± 0.85 |  |
| *Nitrosomonas europaea* | 25 | Whole-cell | 7.0 | 1 | 4000 | 2300 | N/A | [39] |
|  |  |  | 7.5 | 1 | 1600 | 2900 | N/A |  |
|  |  |  | 8.0 | 1 | 480 | 2600 | N/A |  |
|  |  |  | 8.5 | 1 | 300 | 4600 | N/A |  |
|  |  |  | 9.1 | 1 | 140 | 5800 | N/A |  |
|  |  | Cell-free extract | 6.5 | 1 | 10000 | 18000 | N/A |  |
|  |  |  | 7.0 | 1 | 4000 | 23000 | N/A |  |
|  |  |  | 7.5 | 1 | 1300 | 24000 | N/A |  |
|  |  |  | 8.0 | 1 | 320 | 18000 | N/A |  |
|  |  |  | 8.5 | 1 | 120 | 20000 | N/A |  |

^a^*V*_max_ data unavailable for experiment is denoted (N/A)

^b^The individual replicates are plotted in Fig. 4 and Fig. S7

**Table S4.** Cellular morphology of ammonia oxidizers. All cell diameter and lengths were obtained from previously published images. MicrobeTracker [40] was used to calculate cell sizes.

| **Strain** | **Shape^a^** | **Diameter (µm)^b^** | **Length (µm)^b^** | **Surface area / volume ratio** | **Type of images^c^** | **Reference** |
| --- | --- | --- | --- | --- | --- | --- |
| ***Nitrosopumilales* (Group I.1a)** | | | | | | |
| *Nitrosopumilus maritimus* SCM1 | Rod | 0.17-0.22 | 0.5-0.9 | 23.37 ± 2.92 | TEM, SEM | [29, 31] |
| *Nitrosopumilus piranensis* D3C | Rod | 0.2-0.25 [0.22] | 0.49-2.0 [0.85] | 20.53 ± 2.83 | TEM, SEM | [24, 41] |
| *Nitrosopumilus adriaticus* NF5 | Rod | 0.2-0.25 [0.23] | 0.59-1.74 [0.93] | 19.54 ± 2.57 | TEM, SEM | [24, 41] |
| *Nitrosacheum koreensis* MY1 | Rod | 0.3-0.5 [0.35] | 0.6-1.0 [0.6] | 14.76 ± 2.80 | TEM, SEM, n=32 | [20, 32] |
| ‘*Ca.* Nitrosotenuis chungbukensis’ MY2 | Rod | 0.2-0.37 [0.35] | 0.6-1.2 [1] | 13.43 ± 4.91 | TEM, SEM , n=29 | [33] |
| ‘*Ca.* Nitrosotenuis uzonensis’ N4 | Rod | 0.2-0.3 | 0.4-1.7 | 17.9 ± 4.37 | TEM | [1] |
| **‘*Ca.* Nitrosotaleales’ (Group I.1a-associated)** | | | | | | |
| ‘*Ca.* Nitrosotalea devanaterra’ Nd1 | Rod | 0.2-0.34 [0.23] | 0.6-0.94 [0.8] | 19.39 ± 3.9 | TEM, SEM | [30, 34] |
| ‘*Ca.* Nitrosotalea sinensis’ Nd2 | Rod | 0.2-0.34 [0.23] | 0.6-0.8 | 20.25 ± 3.76 | TEM, SEM | [34] |
| ***Nitrososphaerales* (Group I.1b)** | | | | | | |
| ‘*Ca.* Nitrososphaera gargensis’ | Coccus | 0.43-0.46 [0.45] | 0.43-0.46 [0.45] | 13.33 ± 0.38 | TEM, n=4 | [42] |
| *Nitrososphaera viennensis* EN76 | Coccus | 0.5-0.8 | 0.5-0.8 | 9.23 ± 1.85 | TEM, SEM, Phase contrast | [35, 36] |
| ‘*Ca.* Nitrosocosmicus oleophilus’ MY3 | Coccus | 1.0-1.1 [1.1] | 1.0-1.1 [1.1] | 5.45 ± 0.26 | TEM, SEM, n=29 | [38] |
| ‘*Ca.* Nitrosocosmicus franklandus’ C13 | Coccus | 0.43-1.59 | 0.43-1.59 | 5.94 ± 4.38 | TEM, SEM | [18] |
| **Ammonia-oxidizing bacteria** | | | | | | |
| *Nitrosococcus oceani* | Coccus | 1.4-2.2 [1.5] | 1.4-2.2 [1.5] | 4 ± 0.68 | TEM | [43] |
| *Nitrosomonas europaea* | Rod | 0.8-1.1 | 1.0-1.7 | 5.69 ± 0.9 | TEM, Phase contrast | [44, 45] |
| *Nitrosomonas oligotropha* | Rod | 0.5-0.9 [0.6] | 1.1-2.4 [1.7] | 7.84 ± 1.86 | Phase contrast, n=42 | [45] |
| *Nitrosomonas eutropha* Nm57 | Rod | 1.1-1.3 | 1.4-2.6 | 4.48 ± 0.65 | Phase contrast | [45] |
| *Nitrosomonas eutropha* GH22^d^ | Rod | 1.1 | 1.7 | 4.81 |  |  |
| *Nitrosospira briensis* | Spiral | 0.8-1 | 1.5-2.5 | 5.44 ± 0.63 | TEM, SEM | [44, 46] |
| '*Ca.* Nitrosoglobus terrae' | Coccus | 2-3 | 2-3 | 2.4 ± 0.41 | TEM, Phase contrast | [47] |
| ‘*Ca.*Nitrosacidococcus tergens’^d^ | Coccus | 0.5 | 0.5 | 12 | TEM | [48] |
| *Nitrosomonas cryotolerans* | Rod | 1.2-2.2 | 2-4 | 3.02 ± 0.84 | TEM | [49] |
| **Comammox bacteria** |  |  |  |  |  |  |
| *Nitrospira inopinata* | Spiral | 0.2-0.3 | 0.7-1.7 | 17.67 ± 3.44 | TEM, SEM | [19, 50] |

^a^The spiral shaped *N. briensis* and *N. inopinata* were treated as rods.

^b^Mean cell dimesnions that differ from the mean determined by the range are provided in brackets.

^c^TEM, transmission electron microscopy; SEM, scanning electron microscopy; n= number of cells used to calculate mean dimensions if calculated in this study.

^d^The length and diameter of *N. eutropha* GH22 and ‘*Ca.* N. tergens’ are each representative of a single reported value.

**Fig. S1.** **Ammonia oxidation kinetics of *Nitrosopumilales* (Group I.1a) AOA.** Michaelis-Menten plots of *N. maritimus* SCM1, *N. piranensis* D3C, *N. adriaticus* NF5, *N. koreense* MY1, ‘*Ca*. N. chungbukensis’ MY2, and ‘*Ca*. N. uzonensis’ N4. Total ammonium oxidation rates were determined from microsensor measurements of substrate dependent O_2_ consumption from either discrete slopes over many substrate concentrations (a,e,f,h,i,k,o,p,r,v) or a single trace measurement (b,c,d,g,j,l,m,n,q,s,t,u). Apparent half-saturation (*K*_m(app)_) and maximum oxidation rates (*V*_max_) for total ammonium were calculated by fitting the data to the Michaelis-Menten kinetic equation. The red line indicates the best fit of the data. Standard deviations of the estimates based on the non-linear regression are reported. Microrespiration conditions for each strain are presented in Table S2.

**Fig. S2.** **Comparison of AOA apparent substrate affinity (*K*_m(app)_) values across studies.** The (a) *K*_m(app)_ for (NH_3_) and (b) *K*_m(app)_ for total ammonium, of *N. maritimus* SCM1 (brown), *N. koreensis* MY1 (orange), ‘*Ca*. N. uzonensis’ N4 (blue), *N. viennensis* EN76 (purple), *N. inopinata* (green), and ‘*Ca*. N. franklandus’ C13 (yellow) are shown. Symbols filled with light grey represent published values from previous studies and the reference for each strain are indicated. *K*_m(app)_ values were derived from measurements with either pure (circles) or enrichment (diamonds) cultures. Significant differences between the different experiment for each strain was determined by a Student’s *t*-test: **p* < 0.05, ***p* < 0.005, *** *p* < 0.0005.


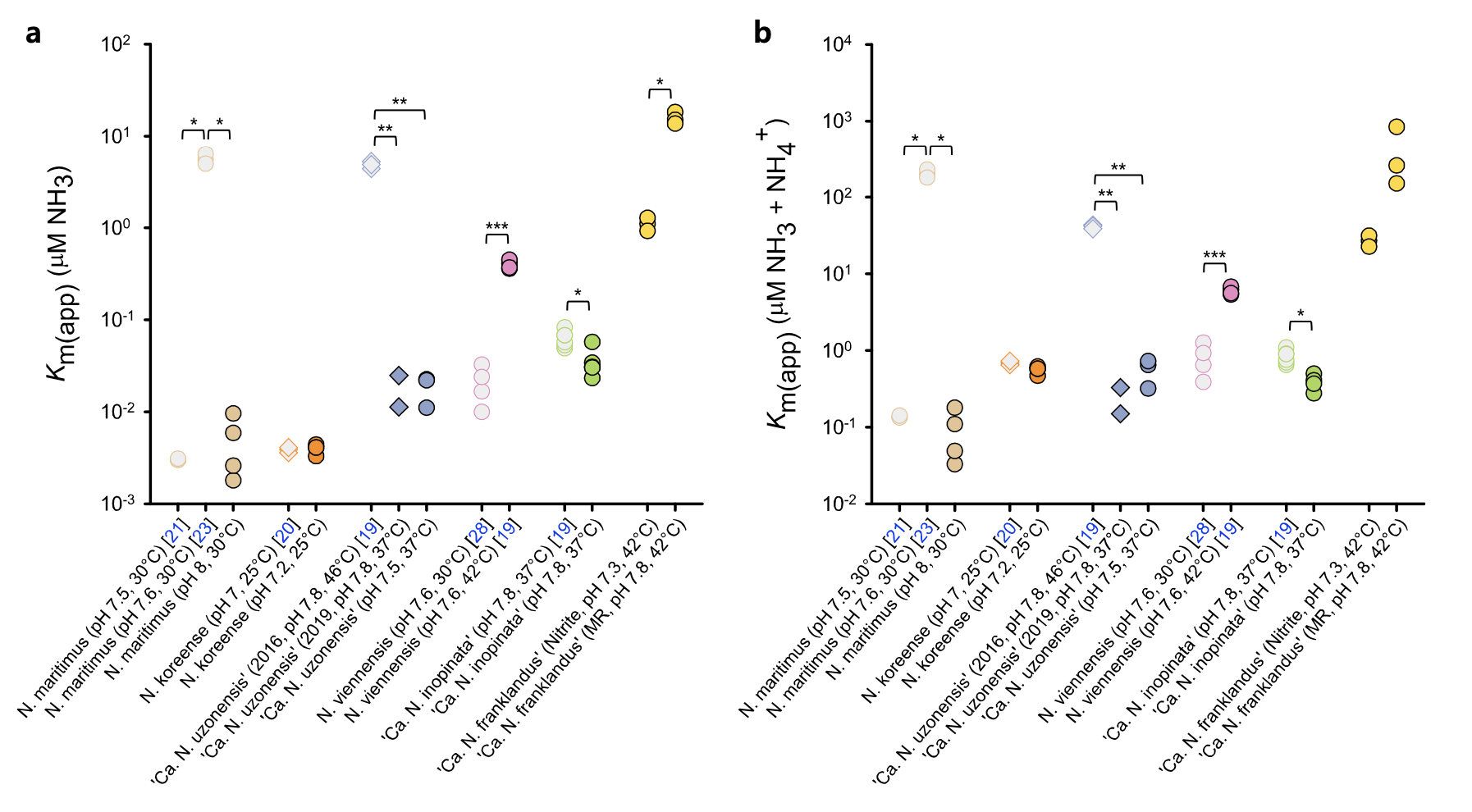


**Fig. S3.** **Ammonia oxidation kinetics of ‘*Ca*. Nitrosotaleales’ (Group I.1a-associated) AOA.** Michaelis-Menten plots for ‘*Ca*. N. devanaterra’ Nd1 and ‘*Ca*. N. sinensis’ Nd2. Total ammonium oxidation rates were determined from microsensor measurements of substrate dependent O_2_ consumption from discrete slopes over many substrate concentrations. Apparent half-saturation (*K*_m(app)_) and maximum oxidation rates (*V*_max_) for total ammonium were calculated by fitting the data to the Michaelis-Menten kinetic equation. The red line indicates the best fit of the data. Standard deviations of the estimates based on the non-linear regression are reported. Microrespiration conditions for each strain are presented in Table S2.

**Fig. S4.** **Ammonia oxidation kinetics of *Nitrososphaerales* (Group I.1b) AOA.** Michaelis-Menten plots for ‘*Ca*. N. nevadensis’ GerE, ‘*Ca*. N. oleophilus’ MY3, and ‘*Ca*. N. franklandus’ C13. Total ammonium oxidation rates were determined from microsensor measurements of substrate dependent O_2_ consumption from either single trace measurements (a-c) or discrete slopes over many substrate concentrations (d-l). Apparent half-saturation (*K*_m(app)_) and maximum oxidation rates (*V*_max_) for total ammonium were calculated by fitting the data to the Michaelis-Menten kinetic equation. The red line indicates the best fit of the data. Standard deviations of the estimates based on the non-linear regression are reported. Microrespiration conditions for each strain are presented in Table S2.

**Fig. S5.** **Ammonia oxidation kinetics of ‘*Ca*. Nitrosocaldales’ (thermophilic) AOA.** Michaelis-Menten plots for ‘*Ca*. N. tenchongensis DRC1’ and ‘*Ca*. N. yellowstonensis’ HL72. Total ammonium oxidation rates were determined from microsensor measurements of substrate dependent O_2_ consumption from discrete slopes over many substrate concentrations. Only the discrete slopes determined with non-inhibitory ammonium concentrations (highlighted with a red box in each panel) were used to calculate kinetic properties. The apparent half-saturation (*K*_m(app)_) and maximum oxidation rates (*V*_max_) for total ammonium were calculated by fitting the data to the Michaelis-Menten kinetic equation. The red line indicates the best fit of the data. Standard deviations of the estimates based on the non-linear regression are reported. Microrespiration conditions for each strain are presented in Table S2.

**Fig. S6.** **Effect of short-term temperature shifts on the cellular kinetic properties of ‘*Ca*. N. oleophilus’ MY3.** The (a) apparent substrate affinity (*K*_m(app)_) for total ammonium, (b) *K*_m(app)_ for NH_3_, (c) the specific substrate affinity (*a*^o^) for total ammonium, (d) the *a*^o^ for NH_3_ and (e) maximum oxidation rate (*V*_max_) determined across a range of temperatures is shown. Duplicate experiments are indicated with black and white circles. All experiments were carried out under identical conditions with a constant pH of 7.3. The average *K*_m(app)_ and *V*_max_ values for ‘*Ca*. N. oleophilus MY3’ at each temperature are provided in Table S3. Temperature sensitivity (*Q*_10_) at temperature range (25–35 °C) was estimated as *Q*10 = *R*_temp + 10_ / *R*_temp_, where *R* is the *K*_m(app)_, *a*^o^, or *V*_max_ at the higher temperature (temp + 10) and lower temperature (temp) [51].

**Fig. S7**. **Microscopic observation of the cell aggregate size of various AOA before (left) and after (right) microrespiration experiments.** DAPI stained (a,b) ‘*Ca.* Nitrosotenuis uzonensis’ N4, (c, d) ‘*Ca*. N. oleophilus’ MY3, and (e, f) *N. piranensis* D3C. Cell aliquots were stained both before (a,c,e) and after (b,d,f) microrespiration experiments. Scale bar 10 µm.

**Fig. S8.** **Ammonia oxidation kinetics of *N. inopinata* and *N. europaea*.** Michaelis-Menten plots for *N. inopinata* (a-d) and *N. europaea* (e,f). Total ammonium oxidation rates were determined from microsensor measurements of substrate dependent O_2_ consumption from either discrete slopes over many substrate concentrations (a,e,f) or a single trace measurement (b-d). Apparent half-saturation (*K*_m(app)_) and maximum oxidation rates (*V*_max_) for total ammonium were calculated by fitting the data to the Michaelis-Menten kinetic equation. The red line indicates the best fit of the data. Standard deviations of the estimates based on the non-linear regression are reported. Microrespiration conditions for each strain are presented in Table S2.

**Supplementary Reference**

1. Lebedeva EV, Hatzenpichler R, Pelletier E, Schuster N, Hauzmayer S, Bulaev A et al. Enrichment and genome sequence of the group I.1a ammonia-oxidizing Archaeon "*Ca.* Nitrosotenuis uzonensis" representing a clade globally distributed in thermal habitats. PLoS One. 2013;8:e80835.

2. Miranda KM, Espey MG, Wink DA. A rapid, simple spectrophotometric method for simultaneous detection of nitrate and nitrite. Nitric Oxide. 2001;5:62-71.

3. Kandeler E, Gerber H. Short-term assay of soil urease activity using colorimetric determination of ammonium. Biol Fert Soils. 1988;6:68-72.

4. Hood-Nowotny R, Umana NH-N, Inselbacher E, Oswald-Lachouani P, Wanek W. Alternative methods for measuring inorganic, organic, and total dissolved nitrogen in soil. Soil Sci Soc Am J. 2010;74:1018-1027.

5. Kits KD, Jung MY, Vierheilig J, Pjevac P, Sedlacek CJ, Liu S et al. Low yield and abiotic origin of N_2_O formed by the complete nitrifier *Nitrospira inopinata*. Nat Commun. 2019;10:1836.

6. **Lane DJ**. *16S/23S rRNA Sequencing: Nucleic Acid Techniques in Bacterial Systematic*. New York: John Wiley and Sons; 1991.

7. Weisburg WG, Barns SM, Pelletier DA, Lane DJ. 16S ribosomal DNA amplification for phylogenetic study. J Bacteriol. 1991;173:697-703.

8. DeLong EF, Taylor LT, Marsh TL, Preston CM. Visualization and enumeration of marine planktonic archaea and bacteria by using polyribonucleotide probes and fluorescent in situ hybridization. Appl Environ Microbiol. 1999;65:5554-5563.

9. Alm EW, Oerther DB, Larsen N, Stahl DA, Raskin L. The oligonucleotide probe database. Appl Environ Microbiol. 1996;62:3557-3559.

10. Amann RI, Binder BJ, Olson RJ, Chisholm SW, Devereux R, Stahl DA. Combination of 16S rRNA-targeted oligonucleotide probes with flow cytometry for analyzing mixed microbial populations. Appl Environ Microbiol. 1990;56:1919-1925.

11. **Bollmann A, French E, Laanbroek HJ**. Chapter three - Isolation, Cultivation, and Characterization of Ammonia-Oxidizing Bacteria and Archaea Adapted to Low Ammonium Concentrations. In: Klotz MG (editor). *Methods in Enzymology*: Academic Press; 2011. pp. 55-88.

12. de la Torre JR, Walker CB, Ingalls AE, Konneke M, Stahl DA. Cultivation of a thermophilic ammonia oxidizing archaeon synthesizing crenarchaeol. Environ Microbiol. 2008;10:810-818.

13. Hedlund BP, Cole JK, Williams AJ, Hou W, Zhou E, Li W et al. A review of the microbiology of the Rehai geothermal field in Tengchong, Yunnan Province, China. Geosci Front. 2012;3:273-288.

14. Hou W, Wang S, Dong H, Jiang H, Briggs BR, Peacock JP et al. A comprehensive census of microbial diversity in hot springs of Tengchong, Yunnan Province China using 16S rRNA gene pyrosequencing. PloS one. 2013;8:e53350.

15. Cole JK, Peacock JP, Dodsworth JA, Williams AJ, Thompson DB, Dong H et al. Sediment microbial communities in Great Boiling Spring are controlled by temperature and distinct from water communities. ISME J. 2013;7:718-729.

16. Kim JG, Park SJ, Sinninghe Damste JS, Schouten S, Rijpstra WI, Jung MY et al. Hydrogen peroxide detoxification is a key mechanism for growth of ammonia-oxidizing archaea. Proc Natl Acad Sci U S A. 2016;113:7888-7893.

17. Wright CL, Schatteman A, Crombie AT, Murrell JC, Lehtovirta-Morley LE. Inhibition of ammonia monooxygenase from ammonia-oxidizing archaea by linear and aromatic alkynes. Appl Environ Microbiol. 2020;86.

18. Lehtovirta-Morley LE, Ross J, Hink L, Weber EB, Gubry-Rangin C, Thion C et al. Isolation of '*Candidatus* Nitrosocosmicus franklandus', a novel ureolytic soil archaeal ammonia oxidiser with tolerance to high ammonia concentration. FEMS Microbiol Ecol. 2016;92:fiw057.

19. Kits KD, Sedlacek CJ, Lebedeva EV, Han P, Bulaev A, Pjevac P et al. Kinetic analysis of a complete nitrifier reveals an oligotrophic lifestyle. Nature. 2017;549:269-272.

20. Jung MY, Park SJ, Min D, Kim JS, Rijpstra WI, Sinninghe Damsté JS et al. Enrichment and characterization of an autotrophic ammonia-oxidizing archaeon of mesophilic crenarchaeal group I.1a from an agricultural soil. Appl Environ Microbiol. 2011;77:8635-8647.

21. Martens-Habbena W, Berube PM, Urakawa H, de la Torre JR, Stahl DA. Ammonia oxidation kinetics determine niche separation of nitrifying archaea and bacteria. Nature. 2009;461:976-979.

22. Prosser JI, Nicol GW. Archaeal and bacterial ammonia-oxidisers in soil: the quest for niche specialisation and differentiation. Trends Microbiol. 2012;20:523-531.

23. Hink L, Lycus P, Gubry-Rangin C, Frostegard A, Nicol GW, Prosser JI et al. Kinetics of NH_3_-oxidation, NO-turnover, N_2_O-production and electron flow during oxygen depletion in model bacterial and archaeal ammonia oxidisers. Environ Microbiol. 2017;19:4882-4896.

24. Bayer B, Vojvoda J, Reinthaler T, Reyes C, Pinto M, Herndl GJ. *Nitrosopumilus adriaticus* sp. nov. and *Nitrosopumilus piranensis* sp. nov., two ammonia-oxidizing archaea from the Adriatic Sea and members of the class *Nitrososphaeria*. Int J Syst Evol Microbiol. 2019;69:1892-1902.

25. Button DK. Biochemical basis for whole-cell uptake kinetics: specific affinity, oligotrophic capacity, and the meaning of the michaelis constant. Appl Environ Microbiol. 1991;57:2033-2038.

26. Prosser JI, Hink L, Gubry-Rangin C, Nicol GW. Nitrous oxide production by ammonia oxidizers: Physiological diversity, niche differentiation and potential mitigation strategies. Glob Chang Biol. 2020;26:103-118.

27. Martens-Habbena W, Stahl DA. Nitrogen metabolism and kinetics of ammonia-oxidizing archaea. Methods Enzymol. 2011;496:465-487.

28. Straka LL, Meinhardt KA, Bollmann A, Stahl DA, Winkler MH. Affinity informs environmental cooperation between ammonia-oxidizing archaea (AOA) and anaerobic ammonia-oxidizing (Anammox) bacteria. ISME J. 2019;13:1997-2004.

29. Könneke M, Bernhard AE, de la Torre JR, Walker CB, Waterbury JB, Stahl DA. Isolation of an autotrophic ammonia-oxidizing marine archaeon. Nature. 2005;437:543-546.

30. Lehtovirta-Morley LE, Stoecker K, Vilcinskas A, Prosser JI, Nicol GW. Cultivation of an obligate acidophilic ammonia oxidizer from a nitrifying acid soil. Proc Natl Acad Sci U S A. 2011;108:15892-15897.

31. Qin W, Heal KR, Ramdasi R, Kobelt JN, Martens-Habbena W, Bertagnolli AD et al. *Nitrosopumilus maritimus* gen. nov., sp. nov., *Nitrosopumilus cobalaminigenes* sp. nov., *Nitrosopumilus oxyclinae* sp. nov., and *Nitrosopumilus ureiphilus* sp. nov., four marine ammonia-oxidizing archaea of the phylum Thaumarchaeota. Int J Syst Evol Microbiol. 2017;67:5067-5079.

32. Jung MY, Islam MA, Gwak JH, Kim JG, Rhee SK. *Nitrosarchaeum koreense* gen. nov., sp. nov., an aerobic and mesophilic, ammonia-oxidizing archaeon member of the phylum *Thaumarchaeota* isolated from agricultural soil. Int J Syst Evol Microbiol. 2018;68:3084-3095.

33. Jung MY, Park SJ, Kim SJ, Kim JG, Sinninghe Damsté JS, Jeon CO et al. A mesophilic, autotrophic, ammonia-oxidizing archaeon of thaumarchaeal group I.1a cultivated from a deep oligotrophic soil horizon. Appl Environ Microbiol. 2014;80:3645-3655.

34. Lehtovirta-Morley LE, Ge C, Ross J, Yao H, Nicol GW, Prosser JI. Characterisation of terrestrial acidophilic archaeal ammonia oxidisers and their inhibition and stimulation by organic compounds. FEMS Microbiol Ecol. 2014;89:542-552.

35. Stieglmeier M, Klingl A, Alves RJ, Rittmann SK, Melcher M, Leisch N et al. *Nitrososphaera viennensis* gen. nov., sp. nov., an aerobic and mesophilic, ammonia-oxidizing archaeon from soil and a member of the archaeal phylum Thaumarchaeota. Int J Syst Evol Microbiol. 2014;64:2738-2752.

36. Tourna M, Stieglmeier M, Spang A, Konneke M, Schintlmeister A, Urich T et al. *Nitrososphaera viennensis*, an ammonia oxidizing archaeon from soil. Proc Natl Acad Sci U S A. 2011;108:8420-8425.

37. Palatinszky M, Herbold C, Jehmlich N, Pogoda M, Han P, von Bergen M et al. Cyanate as an energy source for nitrifiers. Nature. 2015;524:105-108.

38. Jung MY, Kim JG, Sinninghe Damste JS, Rijpstra WI, Madsen EL, Kim SJ et al. A hydrophobic ammonia-oxidizing archaeon of the *Nitrosocosmicus* clade isolated from coal tar-contaminated sediment. Environ Microbiol Rep. 2016;8:983-992.

39. Suzuki I, Dular U, Kwok SC. Ammonia or ammonium ion as substrate for oxidation by *Nitrosomonas europaea* cells and extracts. J Bacteriol. 1974;120:556-558.

40. Sliusarenko O, Heinritz J, Emonet T, Jacobs-Wagner C. High-throughput, subpixel precision analysis of bacterial morphogenesis and intracellular spatio-temporal dynamics. Mol Microbiol. 2011;80:612-627.

41. Bayer B, Vojvoda J, Offre P, Alves RJ, Elisabeth NH, Garcia JA et al. Physiological and genomic characterization of two novel marine thaumarchaeal strains indicates niche differentiation. ISME J. 2015.

42. Spang A, Poehlein A, Offre P, Zumbragel S, Haider S, Rychlik N et al. The genome of the ammonia-oxidizing *Candidatus* Nitrososphaera gargensis: insights into metabolic versatility and environmental adaptations. Environ Microbiol. 2012;14:3122-3145.

43. Klotz MG, Arp DJ, Chain PS, El-Sheikh AF, Hauser LJ, Hommes NG et al. Complete genome sequence of the marine, chemolithoautotrophic, ammonia-oxidizing bacterium *Nitrosococcus oceani* ATCC 19707. Appl Environ Microbiol. 2006;72:6299-6315.

44. Watson SW, Mandel M. Comparison of the morphology and deoxyribonucleic acid composition of 27 strains of nitrifying bacteria. J Bacteriol. 1971;107:563-569.

45. Koops HP, Bottcher B, Moller UC, Pommerening-Roser A, Stehr G. Classification of eight new species of ammonia-oxidizing bacteria: *Nitrosomonas communis* sp. nov., *Nitrosomonas ureae* sp. nov., *Nitrosomonas aestuarii* sp. nov., *Nitrosomonas marina* sp. nov., *Nitrosomonas nitrosa* sp. nov., *Nitrosomonas eutropha* sp. nov., *Nitrosomonas oligotropha* sp. nov. and *Nitrosomonas halophila* sp. nov. J Gen Microbiol. 1991;137:1689-1699.

46. Rice MC, Norton JM, Valois F, Bollmann A, Bottomley PJ, Klotz MG et al. Complete genome of *Nitrosospira briensis* C-128, an ammonia-oxidizing bacterium from agricultural soil. Stand Genomic Sci. 2016;11:46.

47. Hayatsu M, Tago K, Uchiyama I, Toyoda A, Wang Y, Shimomura Y et al. An acid-tolerant ammonia-oxidizing gamma-proteobacterium from soil. ISME J. 2017;11:1130-1141.

48. Picone N, Pol A, Mesman R, van Kessel M, Cremers G, van Gelder AH et al. Ammonia oxidation at pH 2.5 by a new gammaproteobacterial ammonia-oxidizing bacterium. ISME J. 2020.

49. Jones RD, Morita RY, Koops H-P, Watson SW. A new marine ammonium-oxidizing bacterium, *Nitrosomonas cryotolerans* sp. nov. Can J Microbiol. 1988;34:1122-1128.

50. Daims H, Lucker S, Wagner M. A new perspective on microbes formerly known as nitrite-oxidizing bacteria. Trends Microbiol. 2016;24:699-712.

51. Hamdi S, Moyano F, Sall S, Bernoux M, Chevallier T. Synthesis analysis of the temperature sensitivity of soil respiration from laboratory studies in relation to incubation methods and soil conditions. Soil Biology and Biochemistry. 2013;58:115-126.
